# Opinions of maternity care professionals and other stakeholders about integration of maternity care: a qualitative study in the Netherlands

**DOI:** 10.1186/s12884-016-0975-z

**Published:** 2016-07-26

**Authors:** Hilde Perdok, Suze Jans, Corine Verhoeven, Lidewij Henneman, Therese Wiegers, Ben Willem Mol, François Schellevis, Ank de Jonge

**Affiliations:** 1 Department of Midwifery Science, Midwifery Academy Amsterdam/Groningen (AVAG) and EMGO Institute for Health and Care Research, VU University Medical Center, Amsterdam, The Netherlands; 2 Catharina Hospital, Eindhoven, The Netherlands; 3 Department of Clinical Genetics, Section Community Genetics and EMGO Institute for Health and Care Research, VU University Medical Center, Amsterdam, The Netherlands; 4 TNO Quality of Life, Leiden, The Netherlands; 5 Maxima Medical Centre, Veldhoven, The Netherlands; 6 Netherlands Institute for Health Services Research, Utrecht, The Netherlands; 7 Robinson Institute, School of Paediatrics and Reproductive Health, University of Adelaide, Adelaide, Australia; 8 Department of General Practice & Elderly Care Medicine, EMGO Institute for Health and Care Research, VU University Medical Center, Amsterdam, The Netherlands

**Keywords:** Continuity of care, Integrated care, Maternity care, Obstetrics, Midwifery

## Abstract

**Background:**

This study aims to give insight into the opinions of maternity care professionals and other stakeholders on the integration of midwife-led care and obstetrician-led care and on the facilitating and inhibiting factors for integrating maternity care.

**Methods:**

Qualitative study using interviews and focus groups from November 2012 to February 2013 in the Netherlands. Seventeen purposively selected stakeholder representatives participated in individual semi-structured interviews and 21 in focus groups. One face-to-face focus group included a combined group of midwives, obstetricians and a paediatrician involved in maternity care. Two online focus groups included a group of primary care midwives and a group of clinical midwives respectively. Thematic analysis was performed using Atlas.ti. Two researchers independently coded the interview and focus group transcripts by means of a mind map and themes and relations between them were described.

**Results:**

Three main themes were identified with regard to integrating maternity care: client-centred care, continuity of care and task shifting between professionals. Opinions differed regarding the optimal maternity care organisation model. Participants considered the current payment structure an inhibiting factor, whereas a new modified payment structure based on the actual amount of work performed was seen as a facilitating factor. Both midwives and obstetricians indicated that they were afraid to loose autonomy.

**Conclusions:**

An integrated maternity care system may improve client-centred care, provide continuity of care for women during labour and birth and include a shift of responsibilities between health care providers. However, differences of opinion among professionals and other stakeholders with regard to the optimal maternity care organisation model may complicate the implementation of integrated care. Important factors for a successful implementation of integrated maternity care are an appropriate payment structure and maintenance of the autonomy of professionals.

## Background

The way in which maternity care is organized and by whom maternity care is provided shows substantial variation around the world. In a midwife-led care model “the midwife is the lead professional in the planning, organisation and delivery of care given to a woman from initial booking to the postnatal period” [1], in an obstetrician-led care model the obstetrician is the lead professional and in a shared care model the responsibility for the organisation and delivery of care is shared between different health care professionals. The degree of continuity of care is different in each model. In some models the midwife remains the main caregiver after referral to another care provider, whereas in other models the obstetrician takes over responsibility from the midwife entirely when a risk factor or complication occurs.

**Fig. 1:**
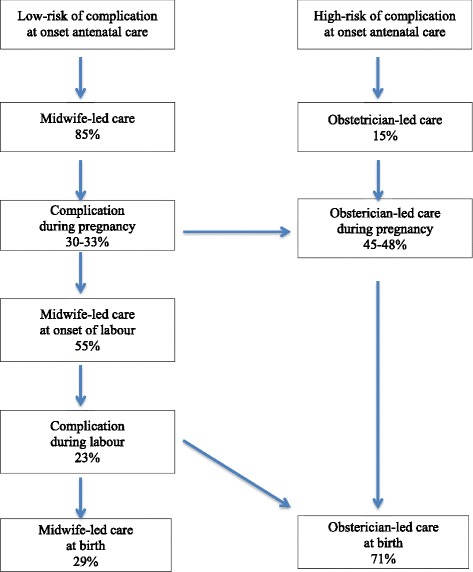
Maternity care in the Netherlands

Maternity care in the Netherlands is organised in two echelons, midwife-led care and obstetrician-led care (Fig. 1), with professionals in these echelons working alongside and complementary to each other. Primary care midwives work autonomously and are responsible for the care of 85 % of women at the start of antenatal care (www.perinatreg.nl/uploads/150/153/PRN_jaarboek_2013_09122014.pdf). Women at low risk of complications, who are in midwife-led care at the onset of labour, may choose to give birth at home or in a hospital. During pregnancy 30 % of women in the Netherlands who start antenatal care with a primary care midwife, develop a risk factor or complication as listed in the national “List of Obstetric indications” [2], and are subsequently referred to secondary or tertiary obstetrician-led care. Responsibility is then taken over by obstetricians and most care is provided by clinical midwives [3]. A primary care midwife no longer has a formal role in the care of women referred to secondary or tertiary care. Of all women in midwife-led care at onset of labour 23 % is referred (www.perinatreg.nl/uploads/150/153/PRN_jaarboek_2013_09122014.pdf). This means that overall approximately two third of *all women* in the Netherlands give birth in an obstetrician-led care setting. Only 0.5 % of women give birth assisted by their general practitioner (http://www.nivel.nl/sites/default/files/Cijfers-uit-de-registratie-van-verloskundigen-peiling-jan-2013.pdf). In the Dutch system, a woman may be transported from home to hospital or from one hospital department to another in case of a referral during labour. Obstetric nurses assist both midwives and obstetricians and provide nursing care during labour in a hospital. Maternity care assistants assist the primary care midwives during labour and care for women at home during the first week after birth.

In the current restricted market-driven health care system in the Netherlands the government is responsible for safeguarding public interests. Health insurers play an active role as health care purchasers and as representatives of their clients’ interests (https://www.nivel.nl/en/governance). The insurance company pays the primary care midwife a fixed fee for care during pregnancy, birth and the postpartum period. This in contrast to the payments of maternity care in hospital, which are not transparent and differ considerably between hospitals [4]. However, the payment structure for maternity care is likely to change as the health insurance companies aim to introduce an overall integrated fee for all maternity care which should be divided among care providers involved.

Although the maternity care system in the Netherlands has been an example for other countries such as Canada [5] the system has also been subject of debate both nationally [6], and internationally [7]. A system with two separate echelons has disadvantages such as discontinuity of care as a result of referrals from midwife-led to obstetrician-led care [8]. Discontinuity of care increases the risk of inaccurate communication [9], and may lead to more interventions and less satisfaction among women [10].

Our previous research in the Netherlands showed that the majority of maternity care professionals are in favour of closer collaboration between primary and secondary care professionals to enhance personal continuity of care for women [11] which was defined as “integrated care” [11]. However, views differ widely on how to operationalise integrated care in practice [11]. In order to improve personal continuity of care, earlier research showed that primary care midwives are willing to expand their tasks to continue management of labour for women that are currently referred to an obstetrician once they have acquired the necessary skills. However, no consensus could be reached on the division of responsibilities and tasks [11]. For innovations in maternity care, such as integration of care, gaining insight into the opinions of health professionals and other stakeholders is important. Innovation strategies can then take these opinions into account.

In the “INtegrated CAre System” study (INCAS), we examined facilitating and inhibiting factors for integration of midwife-led and obstetrician-led care during labour amongst maternity care professionals. This study is the fourth sub-study within the INCAS-study (Fig. 1) [11–13]. The aim of this study is to gain insight into the opinions of maternity care professionals and other stakeholders on the integration of midwife-led care and obstetrician-led care and on facilitating and inhibiting factors for the implementation of this care.

## Methods

A qualitative design was chosen to explore participants’ views and opinions about integrated care in the light of their experience in maternity care. A total of 17 interviews (Table 1) and three focus groups, two of which were online (Table 2), with a total of 21 participants were carried out. Data triangulation was used to enrich the data [14]. Triangulation was achieved by using semi-structured interviews allowing stakeholders to represent their organisations’ opinions and focus groups to explore professionals’ experiences and personal opinions. Data were gathered until saturation was reached. The checklist of the Consolidated Criteria for Reporting Qualitative Research (COREQ) was used when reporting on the data [15]. The study was submitted to the medical ethics committee of VU University Medical Center (reference number 2011/252). An ethical approval was not considered necessary according to the Dutch legislation as this study does not impair medical integrity, it is not stressful for participants and no interventions are performed (https://www.vumc.nl/afdelingen-themas/1646433/7876770/7877361/7955730/nietwmobeslisboom.pdf).

**Table 1:** Participants interviews

Interviews	*n*
Stakeholder representatives (*n* = 17)	
Royal Dutch Organisation of Midwives (KNOV)	1
Dutch Society for Obstetrics and Gynaecology (NVOG)	1
Dutch Organisation for Anaesthesiologists (NVA)	1
Dutch College of General Practitioners (NHG)	1
Dutch Organisation for Maternity Care Assistants (NBvK)	1
Client organisation	2
Health care insurance company	4
Ministry of health	1
Midwifery cooperation	2
Project management organisation in maternity care assistance	1
National collaborating organisation for perinatal care	1
Organization for Health Research and Development (ZonMw)	1

**Table 2:** Focus groups

Focus groups (*n* = 21)	*n*
Focus group Face-to-face (mixed)	
Primary care midwives	2
Clinical midwives	2
Obstetricians	2
Paediatrician	1
Focus group Online	
Primary care midwives	9
Focus group Online	
Clinical midwives	5

### Interviews

A heterogenic group of 17 stakeholders involved in maternity care were purposively selected by the project team for semi-structured interviews, which were held in December 2012. The participants represented different stakeholder organisations and were officially mandated by organisations. (Table 1).

The selected stakeholders all had a professional interest in integrated maternity care or were involved with national or societal discussions related to this topic. All participants were explicitly asked to formulate the viewpoints of their respective organisations. By sending the topic list prior to the interview, participants were able to verify these viewpoints on beforehand if necessary.

### Focus groups

Three focus groups took place between November 2012 and February 2013 (Table 2). Two focus groups were held online. We expected this online methodology to facilitate recruitment, as more professionals might be willing to participate if they were able to join the discussions without traveling and at their own convenience. An independent researcher, not directly involved in maternity care, led all focus groups together with a representative of a client organisation. The face-to-face focus group consisted of two primary care midwives, two clinical midwives, two obstetricians and a paediatrician and were held in a centrally located meeting room. A travel allowance was given to participants.

One online focus group consisted of nine primary care midwives and the other of five clinical midwives.

In our previous study, we found that primary care midwives and clinical midwives have strongly divergent opinions with regards to their responsibilities and tasks [11]. As we were interested in the opinions of both groups, the online focus groups were held for these groups separately. At the time the focus group discussions were conducted, five regions in the Netherlands adopted some type of integrated maternity care. At least one primary and one clinical midwife from each of the five regions with experience in integrated care were invited to participate in the online focus groups.

The face-to-face focus group was tape recorded and fully transcribed. The online focus groups were organised asynchronously using a browser-based application developed by the Netherlands Organisation for Applied Scientific Research, TNO. A new topic (formulated as a question) was introduced online each day during seven consecutive days. Participants could respond 24 h a day, at a time of their own convenience. They were asked to respond to the statement in writing and were encouraged by the moderator to interact with each other. To stimulate active involvement, participants of the online focus group received a gift voucher for books of 25 euro if they responded to all statements at least two times.

The responses of the online focus groups were downloaded.

### Topic list

A multidisciplinary project group consisting of obstetricians, midwives, an obstetric nurse, a paediatrician, a client representative and researchers acted as an advisory panel and approved the topic lists used in the interviews and focus groups. The topic list of the stakeholder interviews (Table 3) was similar to the topic list for the focus group (Tables 4 and 5) and was based on the results of a previous Delphi procedure [11].

**Table 3:** Interview topic guide for stakeholders

Topic
Introduction
Definition of integrated care
• Viewpoint of the organisation.
Knowledge of integrated maternity care
Integration of care within region. Development within own organisation.
Influence of the socio-political context on integration of care
• Does integration of maternity care fit within the political development?
• Does integration of care complement the needs of women?
• Does the media play a role in the development of integrated care?
Characteristics of the organisation
• The ideal structure
• Level of teamwork
• Hierarchy
Collaboration between professionals
• Division of responsibilities
Task-shifting
Characteristics of the adopting person/stakeholder on integration of care
• Do you expect support from your colleagues, other stakeholders or patients?
• Needed competencies
What is needed for successful integration of maternity care?
• Characteristics of innovation (e.g. protocols, finances, education)
How can integrated care be implemented?
Facilitators and inhibitors
Roll of participant’s organisation

**Table 4:** Face-to-face focus group discussion protocol

Topic
Introduction discussion leader and representative of a client organisation
Introduce participants
• Participants are asked for definition of integrated care
• Integration of care within organisation/region
Expectations of maternity care in 10 years
Division of responsibilities in an integrated care system
International best practise
Accepting change; challenges
Successful implementation of integrated care
• What and who is needed
• Facilitators and inhibitors
• Role of professional’s organisation
• Role of insurance companies
Roll of organisation
Questions

**Table 5:** Online focus group topic guide

Day	Topic
1	Reason for integrated care in the region. Most important changes in the region.
	• Facilitators and inhibitors
	Initiator of project
	Expectations
	Collaboration midwives, obstetricians and hospital
2	Changes in care.
	• Changes that have been successful
	• Delegation of tasks
	• Organisation of care
	Finances
3	Experience of integrated care
	• Facilitators/inhibitors integrated care
	Task-shifting
	Experience of clients
	• (Dis) advantages
	Role of clients
5	Responsibilities and competencies of professionals
	• Qualifications of professionals
	Autonomy
6	Requirements for integrated care
	• Personal support
	Additional resources
7	Implementation of integrated care
	• Facilitators and inhibitors
	• Role of professional organisations
	• Role of insurance companies
	Additional points

Fleuren [16] described four categories of determinants based on a literature review and Delphi study among implementation experts that have an important influence on the successful implementation of an innovation: the socio-political context, organization of care, the health care professional and the innovation. We used this model because of its good fit with our study objectives. These categories were included in the topic list for the interviews and focus groups. The topic list consisted of seven semi-structured questions including characteristics of “integrated care” related to previous research [11], specific aspects of integrated care of the participant’s organisation, conditions needed for a successful integration of care and the role that the participant’s organisation could play.

The topic list was sent to the participants by email one week before the interview took place. The semi-structured interviews were carried out by telephone (HP), lasted between 35 and 60 min and were audio recorded. The participants of the focus groups did not receive the topic list beforehand, but the leader used the topic list as a guide.

### Data analysis

Thematic data analysis was used [17]. The interviews were anonymously transcribed (HP, SM). Two researchers (ED, FL) closely read the first two interviews and formulated codes independently, after which they were compared. Consensus on the codes was reached through discussion. The research team, consisting of four researchers including an independent health science expert approved the final coding categories. These were used to code the other interviews (ED, FL). The texts of the focus groups were coded using the same coding categories. Through regular discussion of the findings in the research team, overarching themes were formulated. A frequency analysis of the codes was made. By means of a mind map of the most frequent codes, themes and relations were described. An active search in the data was conducted to find deviant opinions. The software program Atlas.ti version 5.2 was used to support the analysis of the interviews and focus groups discussions. The quotes in the results were translated into English and edited for readability removing words like “uh” without loss of meaning. Characteristics of participants are given in brackets at the end of each quote and are indicated with a number.

## Results

In the face-to-face focus group more discussion and interaction was observed compared to the online focus groups. More comments were made during the discussion with the mixed health professionals by the obstetricians compared to the midwives. The number of reactions on the online forum was 52 responses for the primary care midwives and 46 responses for clinical midwives.

From the interviews and focus group discussions three main themes of integrating maternity care were identified. The first theme was client-centred care with the sub-theme client involvement, collaboration and the type of organisation. The second theme was continuity of care and the third theme was task shifting between professionals with the sub-theme midwifery training.

Facilitating and inhibiting factors for the implementation of integrated care were also identified: the payment structure and professional autonomy. Saturation was reached after seventeen interviews.

### Client-centred care

Most participants agreed that client-centred care is a prerequisite for optimal care, which is the aim of integrating midwife-led and obstetrician-led care. To achieve client-centred care participants indicated that clients must be involved in management of care and decision-making. Moreover, good collaboration between primary and secondary care is needed within an organization: the client should experience a smooth transfer from primary to secondary care.

#### Client involvement

Participants expressed different opinions on the optimal level of client involvement during pregnancy and labour. Opinions varied from freedom of choice for women to limitations prescribed by the professional responsible for medical care, in case of a risk factor.

*“But it is about giving a patient all options, including all risks involved of course. But the patient should be allowed to choose. A patient should decide because it is all about the patient. Sometimes it can be different, it may perhaps be better, medically, to choose another option. But a patient may interpret quality of life differently sometimes. Incomprehensible for a medical professional”. (Representative of a Client organisation, interview #11).*

Participants mentioned a variety of examples with regard to the degree of client involvement for the place of birth and caregiver. Opinions ranged from believing that clients should have the freedom to choose the place of birth based on informed consent, to the opinion that clients should not have a choice in the place of birth at all. Concerning the choice of caregivers, opinions ranged from ‘clients should have complete freedom in choosing their own caregiver’ to ‘caregivers should decide which caregiver should be involved, as clients cannot be held responsible for medical decisions’.

*“I think it’s good to involve the patient but you cannot pass medical responsibility on to the patient. Caregivers must ensure that they can offer a good service. With adequate level of care […]. Trust in the system will then arise”. (Representative of the Dutch College of General Practitioners, interview #2).*

#### Good collaboration

Good collaboration between primary and secondary care was said to be a condition for client-centred care. Several participants agreed that it is important to organise multidisciplinary training for maternity care professionals, to improve collaboration in emergency situations and to have knowledge of each other’s competencies and working methods. According to participants, shared training and more involvement in each other’s working environment could improve communication between professionals as well as improve the quality of care.

*“Yes, I think that if we talk about training, if we would train multidisciplinary, structurally training the whole chain of professionals, that that could have additional value. Occasionally you can see this happening here and there during the “skills and drills training” but this could be very much extended I think”. (Representative of the Royal Dutch Organisation of Midwives, interview #13).*

*“We have gained a better understanding of each others profession: by getting to know the other, trust arises in knowledge and skills. It works two ways: physiology when possible and medical interventions when necessary”. (Primary care midwife, online focusgroup #10).*

#### Type of organisation

Respondents had difficulty describing which type of organization would be ideal in order to provide more client-centred care during labour. However a well operating chain of care was mentioned several times: care in which the various partners work together in a birth centre and the client should experience a smooth transfer. Most participants in both the interviews and focus groups held the opinion that all caregivers should work in the same building and that clients who want to give birth in hospital should not have to be moved to a different department or room when a referral is indicated. According to them, the labour ward must be accommodated and equipped to the needs of both the primary care midwife and the obstetrician.

*“In that type of care [care with division between primary and secondary care], you are still talking about a primary care birth centre where you only carry out primary care things. I do not think that this is the concept of the future because if a woman needs medical attention, which occurs quite often, one has to lug the patient around to another location in the same hospital. I envisage that our care will eventually merge more and more. […] That there should be no door in between, that you can do the transfer from primary to secondary care totally transparent within one open space”. (Representative Project management organisation in maternity care assistance, interview #10)*

One primary care midwife emphasised that the primary care midwifery practices should be part of a larger cooperation to create more efficient collaboration. Participants of one focus group mentioned the need of a team of eight to twelve professionals for the system to function well.

*“We have had some discussions to combine the various midwifery practices into one large [primary care] centre. Antenatal clinics on more than one location, shorter routes for consultation or referral. Choice of place of birth and home birth guaranteed. […]. Joint consultations can subsequently be organized more effectively, as well as training etc. There are a lot of advantages to it, except for the bigger scale”. (Primary care midwife, online focus group #2)*

A counter argument was that if organisations are too big, this could lead to professionals having many meetings at the expense of care for clients.

*“And of course it will be very nice for the College of Perinatal Care to soon be able to say how well everyone is collaborating regionally, but what we see is that it mainly consists of managerial meetings of people who have never seen a postpartum woman before or it has been a long time ago”. (Representative of the Dutch Organisation for Maternity Care Assistants, interview #6)*

### Continuity of care

It was a commonly held view by both maternity caregivers and stakeholders, that continuity of care during labour is important for women in an integrated care system. Although in the current system the primary care midwife only cares for women at low-risk of complications, several participants of both echelons, indicated that primary care midwives should also be the main caregivers after referral during labour so women continue to have the same caregiver. A primary care midwife who already provides this type of care said the following:

*“We conduct regular client satisfaction surveys which show that pregnant women have difficulty with the large number of midwives in our practice … with regards to birth, our pregnant women don’t know any better other than that the midwife will assist them to give birth, and that she has both primary and secondary care responsibilities.*” *(Primary care midwife, online focus group #10).*

Some participants made a distinction between low, moderate and high-risk indications. The following examples of moderate risk indications were given: meconium stained amniotic fluid, need for medical pain relief, prolonged rupture of membranes and a previous caesarean section. Participants stated that primary care midwives could continue to take care of women in labour also when moderate risk situations occur, if necessary after consulting or under supervision of a clinical midwife or obstetrician, leading to a more integrated way of working.

*“I think that you will have to let the ordinary [primary care] midwives support physiology as much as possible and that they will really try their best to really assist people. More nitrous oxide and water injections etc. Doing everything that’s possible with regard to pain relief in primary care. Then the midwife will accompany women and finish things [labours] that are expected to end fairly soon. Meconium, induction of labour and so on…” (Representative of Midwifery cooperation, interview #17).*

*“Maybe formally the obstetrician will remain responsible but the midwife continues to provide care. And more training will be given with regards to surveillance and pathology (Representative of project management organisation in maternity Care Assistance Organisation, interview #10).*

Other participants preferred to adhere to the current system in which the obstetrician takes over the responsibility of care when a moderate or high risk occurs and the primary care midwife assists low risk women remaining skilled in physiological birth. It was noted that the transfer of care must be ‘seamless’ which should be supported by a joint electronic record system and shared protocols.

### Task shifting

According to the majority of participants integration of care will lead to task shifting for all maternity care professionals. This should involve extra training for professionals taking over certain tasks as a condition to obtain new competencies. An example of task shifting is when the “maternity care assistant”, who currently assists the primary care midwife at home during labour would continue to provide assistance to women after referral to hospital. This would mean a shift of tasks from the obstetric nurse to the maternity care assistant for moderate-risk women.

*“I think the maternity care assistant will also have more tasks in the field of risk identification and more coordination with the midwife, and of course providing assistance during labour. […] The maternity care assistant will receive more training in these things and will become more like the obstetric nurse. If the maternity care assistant will be better trained, I think that hospitals will make more use of maternity care assistants during a hospital birth assisted by a primary care midwife”. (Representative of a Health insurance company, interview #8).*

Some midwives mentioned the need for specific competencies such as the ability to interpret continuous electronic fetal heart rate monitoring (EFM). Other participants, however, argued that the primary care midwife should not carry out interventions such as EFM because they would not perform these often enough to guarantee good quality of care.

*“We work with primary care midwives, and they interpret EFM, you know it is all relative and of course it is possible if you have been trained. But I doubt that it is efficient […] you need enough cases and a lot of practice to be good at the secondary care tasks. […] It is about volume of practice. I do not agree with midwives who say they can do both primary and secondary care. We obstetricians have to specialize. Within our team of obstetricians, six of the seventeen have obstetrics as their main field of practise. We try and have one of these six available during every shift. I don’t agree with a midwife saying: “we can all do the same”. Acknowledge what you’re not so good at, and have someone else do that”. (Obstetrician, face-to-face focus group #1).*

#### Midwifery training

Participants agree that training is required if tasks are shifted to other professionals.

Several participants agreed that it is necessary to upgrade midwifery training to an academic level, to be capable of performing more specialised tasks and conducting research. However, some participants prefer to maintain midwifery at a higher professional education level, as they are afraid that upgrading midwifery to a university Master level will be at the expense of hands-on experience of student midwives.

*“I think that you mainly need hands at the bedside and if every midwife is academically educated, I think a lot of power will be lost at the bedside; maybe that is not quite the right word [bedside], in care. […] I think that you disqualify yourself as well by saying that you need an academic education. That would mean that you don’t do it [provide care] well enough at the moment. I do think that they do very well at the moment [provide care]. Rather, you must believe in your own strength, like: we do it our way, and the obstetrician complements that and vice versa”. (Representative of the Ministry of Health, interview #5).*

### Facilitating and inhibiting factors

From the interviews and focus group discussions facilitating and inhibiting factors for the implementation of integrated care were identified. Two factors were found to be most important: the payment structure and professional autonomy.

#### Payment structure

Some participants indicated that the payment structure is a sensitive subject. Participants expressed their concern that in a different payment structure, cost savings could occur which could possibly lead to a reduction in income for health professionals. These concerns may be the cause of resistance to the development of a new funding system.

*“Yes, money, we avoided that a little bit up until now. Yes, but everyone avoids it and at a certain moment you will have to address the issue”. (Obstetrician, face-to-face focus group #1)*

*“Those are things [money] with which people are less willing to take risks. And that starting point makes that it remains a sensitive subject”. (Representative of Midwifery cooperation, interview #9)*

A few participants considered the current financial structure as a threat because referrals from primary care to secondary care or vice versa may be “finance-driven”.

*“At the College of Perinatal Care we are already in favour of an integral payment structure, stemming from the thought that the current system sometimes has incentives for midwives and obstetricians to keep a woman in their care or, say, not return her [to the original caregiver.] It would be better if those incentives no longer existed and that you might have an incentive to collaborate”. (Representative of the National collaborating organisation for perinatal care, interview #12)*

In addition, participants indicated that the influence of health insurance companies should be limited so that optimal care for women can be provided without financial hindrance.

*“Our common goal should be: to give the best care without any form of personal interest or financial drive”. (Representative of the Dutch Society for Obstetrics and Gynaecology, interview #18)*

Opinions on how a new payment structure should be defined differed among participants. A fair distribution of money between care providers based on the actual work performed was said to be important.

#### Professional autonomy

Participants of both the focus groups and interviews indicated the importance of professionals functioning as a team.

*“Both midwives and obstetricians are trained to function autonomously but I hope we can change that into functioning as a team”. (Obstetrician, face-to-face focus group #1).*

*“I think all professionals involved in maternity care are responsible together […] I don’t think you have to lose your own identity”. (Representative of the Royal Dutch Organisation of Midwives, interview #13).*

Professionals are concerned about the loss of autonomy if an integrated care system would be implemented. Most professionals would like to collaborate but wish to remain autonomous when making decisions and in the way they organise their practice.

*“I notice that the Royal Dutch Organisation of Midwives is very frightened of losing part of the autonomy, where it concerns primary care…[…] on the other hand there is a tendency for obstetricians, to say; “if 80 % of women will be in our care sooner or later, let us be in the lead. We can then decide what can be delegated to the midwife”. For midwives that would be the unacceptable” (Representative Project management organisation in maternity care assistance, interview #10)*

Several stakeholders and professionals mentioned that the existing domain struggle between primary and secondary care could be a bottleneck for integration of care. According to participants a joint vision should be formulated and multidisciplinary protocols should be developed, as this would be of benefit to women. Others indicated that it is necessary to formulate the professional organisations’ vision first before making multidisciplinary protocols.

*“You know, the vision of the Royal Dutch Organisation is that in an ideal world we will do all this [making of protocols] together. But it seemed better to us [the KNOV] to first have our own ideas on paper: how we think it should be done. Subsequently, of course you have to talk to your collaborative partners and I understand that the Dutch Society for Obstetrics and Gynaecology will do something similar”. (Representative of the Royal Dutch Organisation of Midwives, interview #13).*

### General characteristics of integrated care

Besides the main themes, participants mentioned the following characteristics of integrated care: a joint electronic client record system for all maternity caregivers, the use of pathways and multidisciplinary protocols supporting a consistent and unequivocal management of care in primary and secondary care for women, mutual respect among professionals, intakes for pregnant women jointly by midwives and obstetricians, a buddy system between obstetricians and midwifery practices for more collaborative work and consultations by obstetricians in midwifery practises as opposed to consultations after referral to hospital.

## Discussion

The aim of this study was to gain insight into the opinions of maternity care professionals and other stakeholders on the integration of midwife-led care and obstetrician-led care and on facilitating and inhibiting factors for the implementation of this care. For most professionals it appeared to be difficult to envisage a system, which does not yet exist and to think “out of the box”. Nonetheless, client-centred care and continuity of care for women were found to be important characteristics of an integrated maternity care system by participants. Opinions differed regarding the optimal maternity care organisation model. Participants indicated that inhibiting factors for integrating maternity care are the payment structure and fear of losing autonomy.

In this study we explored the relevant topics for our maternity care model, which is in the process of change. Other studies have not explicitly explored opinions regarding integrated care at both professional and management level. The combination of interviews and focus groups enhances trustworthiness of findings, making the results more robust. The interviews and focus groups generated a broad range of opinions regarding integrated care, giving a realistic impression of opinions in the whole field [14] at both professional and management level. The stakeholders were asked not to give their personal opinion but that of the organisation they represented. We realise that this was sometimes difficult for participants. The advantage of bringing together a diverse group of professionals for our face-to-face focusgroup was that it maximized exploration of different perspectives. However, hierarchy may have affected individual participants [18]. Since obstetricians made more comments compared to the midwives during the face-to-face focusgroup, this may have been the case in this study causing overestimation of the weight of themes. Because the interviews were carried out by telephone and two focus groups were carried out online, it was not possible to observe body language of the participants. The fact that interaction amongst participants in the online focus groups was limited may have been the result of participants not meeting face-to-face. This could also have been the result of not being able to supervise the discussions 24 h a day and respond to participants immediately.

The current restricted market-driven health care system in the Netherlands might strengthen the individual interests of professionals, instead of stimulating collaboration between professionals to achieve optimal care (http://www.nza.nl/104107/139830/465987/Advies_Bekostiging_-integrale-_zorg_rondom_zwangerschap_en_geboorte.pdf). If a woman is referred to the obstetrician during pregnancy, remuneration could be the “trigger” to keep her in obstetrician-led care and vice versa, finances could “trigger” midwives to take care of women longer than they would if finances did not play a part. Participants in our study and in the study of Avery et al. [19] recognize the “finance driven competition for clients” and agree that this must be changed in a modified system, as this does not help to achieve optimal care for clients. However, opinions differ with regards to the design of a new payment structure from an integrated tariff to separate tariffs for each professional group, which could be nationally or regionally determined. Participants agree that health insurance companies should not be allowed to have a major role in determining care policy. To support a decision on the best maternity care system and one that is economically feasible, the Dutch Healthcare Authority (NZA) has stimulated experiments in pilot regions, which are currently being carried out (http://incas2.nl/INCAS-2/).

Participants in our study state that the two separate echelons, which currently exist, may have disadvantages with regards to continuity of care. In countries such as Canada (http://www.canadianmidwives.org/DATA/DOCUMENT/CAM_ENG_Midwifery_Care_Normal_Birth_FINAL_Nov_2010.pdf) and New Zealand [20] midwives move between primary and secondary care settings leading to more personal continuity of care for women. All participants in this study mentioned that personal continuity of care for women is important. This is consistent with earlier findings showing that there is consensus among professionals to minimize the number of professionals involved during labour [13] clients appreciate the continuity of care given by a primary care midwife after referral [21], and clients rate the quality of care higher if they know their care provider prior to going into labour [22]. In our study participants pointed out that this can be achieved if the primary care midwife could remain the caregiver for women with a moderate risk indication, with or without consulting a clinical midwife or obstetrician. However, to realise continuity of care, task shifting is needed but this in itself can be seen as an inhibiting factor for integrating care. Both this study and prior research [11] show a lack of agreement among maternity care professionals with regard to task shifting.

Participants in our study mentioned client-centred care as an important basis for a maternity care model. However, it remains unclear how patient preferences should be balanced with physicians’ opinion [23]. In line with this, our study shows that tension exists between professionals related to the level of client involvement in maternity care, which ranges from the opinion that all decisions should be made by the client to the other extreme that the professional decides what is best for the client. Our results are in accordance with literature [24] showing that opinions of maternity care experts are divided with regards to the amount of professional advice that should be given to women. Our suggestion is that professionals in the Netherlands give more information specifically tailored to each individual woman and move to a model of Shared Decision Making (SDM) which has been shown to have a positive impact on the childbirth experience [25]. SDM is defined as “an approach where the clinician and client share the best available evidence when faced with the task of making decisions, and where the client is supported to consider options, to achieve informed preferences” [26]. Open and respectful communication between women and care professionals will help practitioners in SDM [24]. As well as supporting the client, a common orientation towards the client is beneficial for the success of interdisciplinary teams [27].

Different regions in the Netherlands already have experience with some form of “integrated care”. For example, in one region, the primary care midwife continues to care for women with moderate risk indications during birth [28]. Midwives in our study indicated that although the workload was high in this region, work satisfaction was even greater and satisfaction among clients was very high compared to other regions. In contrast, obstetricians in our study held the opinion that further specialisation is needed among all professionals to increase volume of practice and ensure optimal quality of care. However, literature shows that professionals might best serve the client by providing continuity of care [21] and having common goals and visions among professionals [29].

The current training of midwives and obstetricians is completely separate in the Netherlands. However, participants indicated that some combined education for maternity care professionals would be better to share ideas and broaden their horizon. The importance of interprofessional training is widely accepted [19, 30] and can help develop professional competence, a joint attitude towards the client, interprofessional respect [27] and better teamwork, which could improve quality of care for women.

In contrast to countries such as Canada and New Zealand (http://www.canadianmidwives.org/midwifery-education.html; https://www.midwife.org.nz/education) where midwifery training leads to a university degree, midwifery education in the Netherlands is at higher professional education level. Several participants agreed that it is necessary to upgrade midwifery training in the Netherlands. A few participants were sceptical about this, as they fear that the “practical hands-on midwifery” could disappear. However, general practitioners in the Netherlands have a history of upgrading their level of practice from a Bachelor to a Master’s Degree. This change in academic education has enabled them to support their clinical practice with scientific evidence [31], which strengthened the profession. Currently, the Midwifery Academies are developing a new curriculum at Master’s Level (www.verloskunde-academie.nl/academisering/). Training at academic level will enable the midwife to be a strong advocate for clients by translating scientific evidence in a way that enables pregnant women and their partners to make the choices that are right for them. An academic partner for obstetricians could facilitate the integration of maternity care.

Autonomy is considered to be very important by midwives and obstetricians because they do not want to lose control and independence in their clinical decision-making. In the current maternity care system collaboration already exists between the two professions although they are autonomous in making decisions regarding the management of care. Midwives and obstetricians in this study and professionals in our previous Delphi study [11] expressed fear of losing this autonomy if maternity care is integrated. This is complicated by the fact that professionals disagree about role boundaries [11, 29]. In addition, deep-seated philosophical differences about childbirth generate tensions [32]. In this study participants agreed that to improve good collaborative practice between midwives and obstetricians, respect and accountability are essential as well as clearly identified responsibilities for the different professionals. In the UK, stricter delineation of the boundaries between midwifery and obstetrics increased the confidence and professional visibility of midwives but left doctors feeling excluded and undervalued [33]. In order to achieve good collaborative practice, instead of mainly focussing on autonomy, the skills and qualities that form the basis of “professional courtesy” need to be recognised in one another [32].

Since the start of the data collection in this study, several regions in the Netherlands have already initiated some form of integrated care but a lot of regional variation does exist. A follow-up study is on-going in which these regions will be valuated in terms of clinical outcomes, experiences of women and professionals and costs. By comparing outcomes and experiences between regions, lessons can be learned about the optimal model of integrated care.

## Conclusions

Maternity care professionals and other stakeholders who participated in this study indicated that the optimal maternity care system should be client-centred, provide continuity of care for women during labour and birth and include a shift of responsibilities between health care providers. However, opinions differed with regard to the optimal maternity care organisation model, which could complicate the implementation of integrated care.

Important factors for a successful implementation of integrated maternity care are an appropriate payment structure and maintenance of the autonomy of professionals. These factors need to be addressed when implementing an integrated maternity care system.
